# Region-Based Segmentation and Wiener Pilot-Based Novel Amoeba Denoising Scheme for CT Imaging

**DOI:** 10.1155/2020/6172046

**Published:** 2020-12-13

**Authors:** Syed Muhammad Umar Talha, Tariq Mairaj, Waleed Bin Yousuf, Jawwad Ali Zahed

**Affiliations:** ^1^Department of Electrical Engineering, Pakistan Navy Engineering College, National University of Sciences and Technology, H-12 Islamabad, Pakistan; ^2^Department of Telecommunication Engineering, Sir Syed University of Engineering & Technology, Karachi, Pakistan

## Abstract

Computed tomography (CT) is one of the most common and beneficial medical imaging schemes, but the associated high radiation dose injurious to the patient is always a concern. Therefore, postprocessing-based enhancement of a CT reconstructed image acquired using a reduced dose is an active research area. Amoeba- (or spatially variant kernel-) based filtering is a strong candidate scheme for postprocessing of the CT image, which adapts its shape according to the image contents. In the reported research work, the amoeba filtering is customized for postprocessing of CT images acquired at a reduced X-ray dose. The proposed scheme modifies both the pilot image formation and amoeba shaping mechanism of the conventional amoeba implementation. The proposed scheme uses a Wiener filter-based pilot image, while region-based segmentation is used for amoeba shaping instead of the conventional amoeba distance-based approach. The merits of the proposed scheme include being more suitable for CT images because of the similar region-based and symmetric nature of the human body anatomy, image smoothing without compromising on the edge details, and being adaptive in nature and more robust to noise. The performance of the proposed amoeba scheme is compared to the traditional amoeba kernel in the image denoising application for CT images using filtered back projection (FBP) on sparse-view projections. The scheme is supported by computer simulations using fan-beam projections of clinically reconstructed and simulated head CT phantoms. The scheme is tested using multiple image quality matrices, in the presence of additive projection noise. The scheme implementation significantly improves the image quality visually and statistically, providing better contrast and image smoothing without compromising on edge details. Promising results indicate the efficacy of the proposed scheme.

## 1. Introduction

Computed tomography (CT) has served as a fundamental tool for human internal anatomy visualization since its development and subsequent commercialization; with considerable benefits for mankind [1–6]. In radiology, CT uses projections from multiple cross-sectional views; consequently, patients are subjected to extensive radiation exposure. Although CT is a very informative medical diagnosis tool, the high radiation dose is a concern due to the associated hazards [2, 7, 8]. However, the reduction of radiation dose degrades the reconstructed image quality in most commonly used CT reconstruction algorithms. Developing techniques for CT reconstruction with reduced radiation dose is an active research area. Various strategies have been adopted to reduce the radiation dose, such as automatic exposure control, adjusting kV with respect to the patient or organ-specific dose, protocol optimization, postprocessing, advanced reconstruction techniques, limited data, and few-view techniques, etc. [2, 8–16]. Many advanced CT scanner designs have also been developed to support dose reduction, such as interior CT, low-dose CT, and sparse-view CT [17–19].

Sparse-view CT is a particular type of CT scans where the number of data acquisition views is reduced, while keeping the X-ray tube current at the standard level. The sparse-view CT benefits in reducing radiation dose and scan time in cardiac CT [17]. Moreover, other sparse-view CT applications are micro-CT (for small animal imaging), flat panel detector, 3D angiography, and other industrial CT applications [17, 19–21]. However, conventional CT reconstruction algorithms, such as filtered back projection (FBP), are designed for densely sampled angular projections. Therefore, such algorithms do not produce diagnostic quality reconstructed images in sparse-view CT. The features in the reconstructed images can be enhanced through postprocessing; in which image processing techniques are widely used. Sophisticated image processing techniques offer a potential to extract more enhanced features from the reconstructed CT images.

Amoeba-based filtering [22] schemes have been a revolutionary contribution in image filtering applications. Amoeba is a spatially variant filter kernel which adapts its shape according to the image contents, intending to preserve edge details and contour boundaries while smoothing the degraded image segments. Amoeba-based filtering and its variants have been applied in many image processing applications such as adaptive neighborhood morphology (Debayle and Pinoli 2005), bilateral structuring functions (Angulo 2011), salience adaptive structuring elements (Curic et al. 2012), nonlocal patch-distance-based amoebas (Yang and Li 2015), and haze removal (Zhang and Wei 2019) [23–27]. The adaptive nature of amoeba-based filtering [22] makes it efficient in many dynamic applications, such as range imaging, medical imaging, segmentation, and image denoising, For CT images, some prior knowledge of image exists because of human body anatomy and its symmetry and contagious nature of pixel spread, which allows amoeba to extract missing information from the reconstructed image [28, 29].

In this reported work, the amoeba-based image filtering is customized for CT images acquired through filtered back projection (FBP) using sparse projection data. The proposed and customized amoeba scheme enhances the quality of the degraded sparse-view FBP reconstructed image. The amoeba kernel is derived from a pilot image based on the Wiener filter. The Wiener filter is much superior in image denoising and restoration than many other techniques, such as simple inverse filtering, Gaussian filtering, and mean filtering [30]. Therefore, the Wiener filter-based pilot image suppresses noise while preserving the image details, unlike the Gaussian-based pilot in classical amoeba and its variants [22, 26, 30]. Furthermore, the presented work also improves the methodology for amoeba shape acquisition by replacing the classical amoeba distance-based approach with region-based segmentation. The scheme is implemented using simulated and clinically reconstructed head CT phantoms. The proposed scheme is applied to noisy sparse projection data, and the reconstructed image quality is also investigated using multiple image quality metrics. Amoeba-based filtering is customized such that the image similarity index improves at even a lesser radiation dose.

The detail of the proposed research methodology and case study is given in the next section, followed by Results and Discussion, and then by Conclusion.

## 2. Methodology

Many modern reconstruction techniques, including advanced reconstruction techniques, use sophisticated postprocessing/image processing techniques for better representation of the reconstructed image. The dynamic and adaptive nature of Amoeba filtering makes it a strong candidate for CT application. Amoeba filtering (Lerallut et al. 2007) [22] is a particular case of spatially variant image filtering, which considers the image gradient for determining the amoeba kernel shape. The classical amoeba filtering is dependent upon the amoeba distance-based approach for the growth of amoeba [22]; this generic approach is generally efficient in most natural images. However, CT images are generally divided into various regions, based on human anatomy and test phantoms. The proposed scheme uses region-based segmentation (RBS) for amoeba shaping mechanism; hence, it is more related to the CT problem and solves it more aptly as compared to the classical amoeba.

It is noteworthy that the amoeba kernel is derived from a pilot image. Classical amoeba filters [22] and its variants predominantly use a large Gaussian filter to create the pilot image. Although the Gaussian pilot ensures significant smoothing; however, it still contains significant noise which limits the growth of the amoeba body. To offer improved enhancement of scans, a scheme comprising of Wiener filter-based pilot image and region-based segmentation (RBS) for amoeba kernel shaping is proposed.

### 2.1. Wiener Filter-Based Pilot Image

As the shape of the amoeba is dependent upon the center pixel of the sliding window, it is essential that the amoeba is not wrapped around a noisy pixel. As a remedy, the amoeba shape is calculated from a pilot image instead of the degraded reconstructed image. Wiener filter is very efficient in image denoising and restoration applications as it uses image degradation function and noise statistics [30]. The Wiener filter is adaptively applied to the degraded reconstructed image. The applied smoothing depends upon the image variance; the greater the variance, the lesser the smoothening or vice versa [30, 31]. Thus, the pilot image, so formed has minimal image degradation and improved edge preservation.

The Wiener filtered image (*W*) applied to degraded image intensity *D*, with all pixels *p* = 1: *P*, is given as

(1) $$\begin{array}{c} W\left(p\right)=\mu_{L}\left(p\right)+\left(\frac{\sigma_{L}^{2}\left(p\right)-{\sigma_{n}}^{2}}{\sigma_{L}^{2}\left(p\right)}\right)\left(D\left(p\right)-\mu_{L}\left(p\right)\right), \end{array}$$

where each pixel index *p* is represented with (*x*, *y*)|_*x*=1:*X*;*y*=1:*Y*_, *σ*_*n*_^2^ is the noise variance of the degraded image *D*, and *μ*_*L*_ and *σ*_*L*_^2^ represent the mean and variance, respectively, in a 2D sliding window about pixel *p*, computed using

(2) $$\begin{array}{c} \mu_{L}\left(p\right)=\frac{1}{UV\;}\overset{U}{\underset{u=1}{\sum }}\;\overset{V}{\underset{v=1\;}{\sum }}I_{p}\left(u,v\right), \end{array}$$

(3) $$\begin{array}{c} \sigma_{L}^{2}\left(p\right)=\left[\frac{1}{UV\;}\overset{U}{\underset{u=1}{\sum }}\;\overset{V}{\underset{v=1}{\sum }}I_{p}^{2}\left(u,v\right)\right]-\mu_{L}^{2}\left(p\right), \end{array}$$

where *I*_*p*_(*u*, *v*) is the matrix containing intensity values within the local neighborhood about pixel *p*(*x*, *y*, and *U* and *V* define the size of the 2D sliding window with running indices *u* and *v*.

### 2.2. Proposed Amoeba Filter Kernel Using RBS (Region-Based Segmentation)

The proposed amoeba adapts its shape with respect to the internal image contours and edges. The amoeba filter uses the classical sliding window model for image filtering, where the window is centered at each pixel of the image. The shape of the amoeba kernel is initialized as the entire sliding window (square), which then adopts the shape based on window contents. The amoeba shaping algorithm is inspired by automated segmentation applications and techniques, such as active contours models and its variants [22, 32–34]. The proposed amoeba shaping uses region-based segmentation (RBS) through multilevel thresholding [35–38].

**Figure 1:**
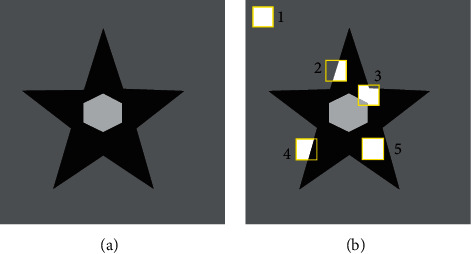
Self-shaping amoeba based on the location of sliding window. (a) Sample image. (b) Initially, square shape kernel is modified at different spatial locations within the image.

In the proposed amoeba shaping mechanism, a “region” is referred to a cluster of contagious pixels, of uniform/near-uniform image intensities, inside the sliding window. The surrounding contour is automatically detected by the proposed algorithm. The amoeba shape remains square on sliding windows with little/no image intensity variation. However, with multiple variations in intensities, amoeba takes the shape of the region containing the window-center pixel. The amoeba shaping process is illustrated on a test image in Figure 1. The image contains three intensity levels. Different instances of the rectangular sliding window are represented by a (yellow) rectangle outlined in Figure 1(b), whereas the different adopted amoeba shapes in the windows are shown in white color. The two rectangular shaped amoebas 1 and 5 contain no variation. However, 2, 3, and 4 amoebas take nonrectangular shapes of the region containing center pixel.

The sliding window, *I*_*p*_(*u*, *v*), around the pixel *p*(*x*, *y*) contains ‘*N*' regions (*R*_1_ : *R*_*N*_) given as

(4) $$\begin{array}{c} \overset{N}{\underset{n=1}{⋃}}R_{n}=I_{p}\left(u,v\right)\;|\;R_{n1}\cap R_{n2}=\emptyset \;\forall n1\neq n2, \end{array}$$

where region *R*_*n*_ is the *n*^th^ segmented region inside the sliding window *I*_*p*_(*u*, *v*). Image histogram (*H*) of *I*_*p*_(*u*, *v*) is computed, and the gray levels are classified into multiple bins or clusters based on adaptive multilevel thresholding. Discontinuities, interclass variations, peaks, and valleys in the histogram are used to determine adaptive multilevel thresholds (*T*_1:*N*_). Various adaptive multilevel thresholding techniques are available in the literature [39–43]. This reported work uses Otsu's method [42, 43], which is a widely used technique in computer vision and image processing applications. Otsu's method sets the optimal threshold levels as the values which maximize the variance between classes or bins [42, 43]. A bin of interest is determined by the threshold levels containing the intensity of the window center, *p*(*x*, *y*). The segmented binary image mask determining the pixels included in the bin of interest (*B*_in_) is given as

(5) $$\begin{array}{c} B_{\text{in}}\left(u,v\right)=\left\{\begin{array}{c} 1\text{ if }T_{\text{in}-1}<I_{p}\left(u,v\right)\leq T_{\text{in}}\\ 0\text{ otherwise} \end{array}\right.\;|\;p\left(x,y\right)\in B_{\text{in}}, \end{array}$$

where *T*_in−1_ and *T*_in_ are the corresponding lower and upper threshold limits determining the bin of interest *B*_in_. Some subregions inside *B*_in_ may be noncontagious, so only a subregion containing center pixel is used in final amoeba shape formation. This is manifested using connected components labeling, a common technique in image processing and computer vision applications for labeling and extracting image disjoints [30, 44, 45]. Details along with the algorithm representation and pseudocode are given in appendix A and B, respectively.

The final amoeba kernel shape, *A*(*u*, *v*), is constituted of the pixels making up the central subregion containing the central pixel *p*(*x*, *y*) in the sliding window or local neighborhood (*U*, *V*). The same process is repeated for each sliding to find the corresponding amoeba kernel shapes. Mean operation is then applied on each final amoeba kernel shape to compute the intensity of the output image at the corresponding central pixel.

**Figure 2:**
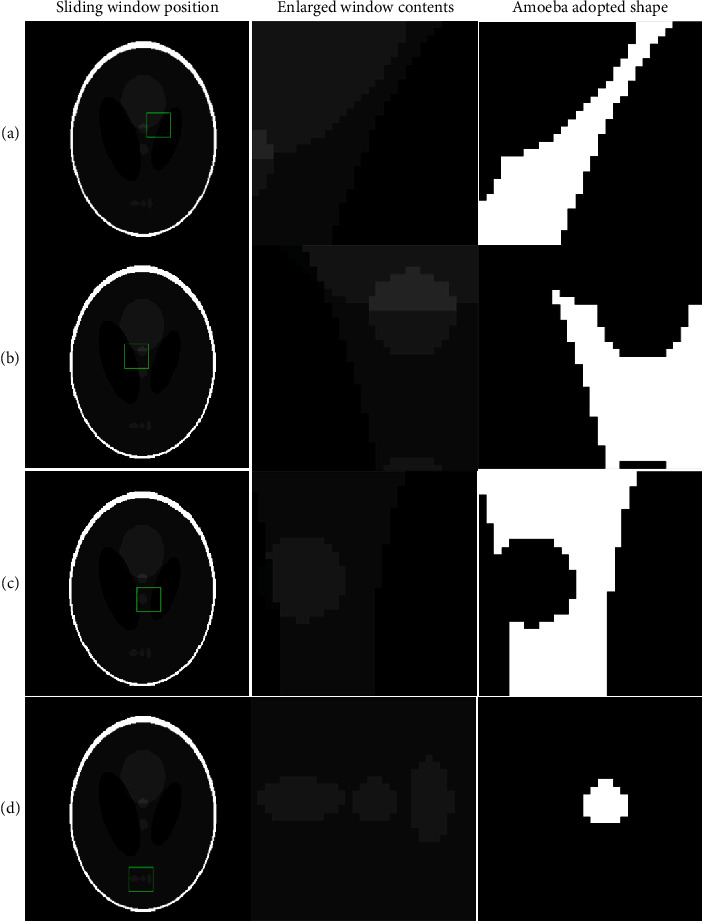
Adopted amoeba kernel shape at various spatial location of sliding window. Each row (a–d) contains a sample of sliding window at a spatial location, along with an enlarged view of the window content and the adopted amoeba shape.

The effectiveness of the proposed self-shaping amoeba filter kernel in computed tomography applications is showcased in Figures 2(a)–2(d) using the Shepp-Logan CT phantom (512 × 512 pixels). Four different instances of shapes adapted by the amoeba depending upon the spatial location of the sliding window are displayed in each of rows (a) to (d) of Figure 2. Column 1 in each row (Figures 2(a)–2(d)) demonstrates the location of the sliding window in the image; column 2 displays the zoomed contents of the sliding window. White regions in column 3 are representing the adapted amoeba filter kernel shapes. Figure 2 uses a sliding window of 50 × 50 pixels for better visualization of amoeba shaping.

### 2.3. Amoeba Filtering-Based CT Image Enhancement

**Figure 3:**
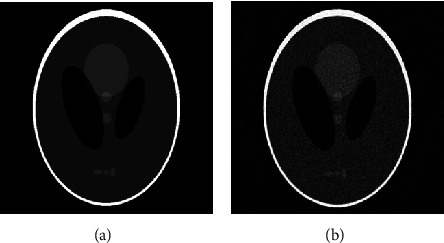
Sparse-view FBP reconstruction of simulated phantom (a) Shepp-Logan phantom. (b) 2-degree angularly sampled FBP reconstruction of (a).

The most basic and common head CT phantom, Shepp-Logan, was used in this implementation as the subject to acquire the projection data. It is a standard test image and synthetic phantom. The size of the head phantom was kept as 512 × 512 pixels, as shown in Figure 3(a). Ninety fan-beam projections were taken from view angles 0 to 180 degrees; with an angular sampling interval of 2 degrees. The detector sensors were spaced at 0.25 mm.

The acquired projection data contains noise from numerous factors including photons, quantization, and electronics. In the literature, various medical imaging techniques use different types of noise models in which the Gaussian model is commonly used for CT [46–49]. Therefore, in the proposed work, additive white Gaussian noise (AWGN) at 3 dB SNR level is used. Filtered back projection (FBP) reconstruction algorithm is then applied to the projection data, which is the most commonly used CT reconstruction algorithm [8, 50, 51]. The resultant FBP reconstructed image is of low quality and is severely degraded (due to reduced dose), as shown in Figure 3(b).

The smoothness of the reconstructed image was lost due to undersampled FBP reconstruction. The amoeba filtering was used for smoothening without compromising on edge details. The size of the sliding window was kept as 9 × 9 pixels for the pilot image and amoeba shaping, through the mechanism described in the previous section. Once the amoeba shape is determined, the mean filtering was applied.

**Figure 4:**
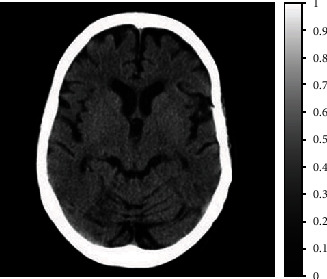
Clinically reconstructed head CT phantom acquired from Phillips CT healthcare case study, available at [52].

The proposed amoeba denoising scheme was also implemented to clinically reconstructed CT head image from Phillips CT healthcare case study, available at [52] as shown in Figure 4. The size of the reconstructed head phantom was kept at 512 × 512 pixels. The sparse-view projections were acquired at an angular sampling of 2 degrees, with AWGN added in projections at 3 dB SNR. The pilot image for amoeba shaping was acquired using a Wiener filter with a kernel neighborhood of size 9 × 9 pixels.

### 2.4. Image Quality Metrics

The quality of the scheme was evaluated using multiple full-reference objective image quality metrics [53]. The image quality metrics use the distortion-free original image/phantom as a reference. The image quality metrics include RMSE (root mean square error), PSNR (peak-signal-to-noise ratio), SSIM (structural similarity index metric), EPI (edge preservation index), SI (sharpness index), SC (structural content), and NAE (normalized absolute error).

RMSE is computed by taking the square root of the average of squares of the differences of the corresponding pixels in the test and reference images and is given as

(6) $$\begin{array}{c} \text{RMSE}=\sqrt{\;\frac{1}{m\;n\;}\overset{m-1}{\underset{i=0}{\sum }}\overset{n-1}{\underset{j=0}{\sum }}{\left[A\left(i,j\right)-B\left(i,j\right)\right]}^{2}}, \end{array}$$

where *A* is the reference image, *B* is a test image, and *m* and *n* define the size of the reference and test images.

The PSNR is expressed in terms of decibels (dB), calculated as

(7) $$\begin{array}{c} \text{PSNR}=20\;\log10\left(\frac{I_{\max}}{\text{RMSE}}\right), \end{array}$$

where *I*_max_ is the maximum pixel intensity value in the image.

PSNR and RMSE use pixel intensity differences to evaluate the image quality, which although have clear mathematical and physical significance but offer very less in terms of human visual perception of image quality [53, 54], whereas SSIM incorporates the luminance, contrast, and structural details and similarity of the compared images, giving much appropriate representation of the human preserved visual image quality [53, 54]. These terms, namely, the luminance index (li), the contrast index (ci) and the structural index (si); combines to determine the SSIM value as

(8) $$\begin{array}{c} {\text{SSIM}}_{\left(x,y\right)}={\left[{\text{li}}_{\left(x,y\right)}\right]}^{\propto }.{\left[{\text{ci}}_{\left(x,y\right)}\right]}^{\beta }.{\left[{\text{si}}_{\left(x,y\right)}\right]}^{\gamma }, \end{array}$$

where

(9) $$\begin{array}{c} {\text{li}}_{\left(x,y\right)}=\frac{2\mu_{x}\mu_{y}+C_{1}}{\mu_{x}^{2}+\mu_{y}^{2}+C_{1}}, \end{array}$$

(10) $$\begin{array}{c} {\text{ci}}_{\left(x,y\right)}=\frac{2\sigma_{x}\sigma_{y}+C_{2}}{\sigma_{x}^{2}+\sigma_{y}^{2}+C_{2}}, \end{array}$$

(11) $$\begin{array}{c} {\text{si}}_{\left(x,y\right)}=\frac{\sigma_{xy}+C_{3}}{\sigma_{x}\sigma_{y}+C_{3}}, \end{array}$$

where *μ*_*x*_ and *μ*_*y*_ are intensity means along the *x* and *y* directions, *σ*_*x*_ and *σ*_*y*_ are image standard deviations, and *σ*_*xy*_ is the cross-covariance of images in *x* and *y* directions [53]. *C*_1_, *C*_2_, and *C*_3_ are small constant terms added to avoid instability [53]. *α*, *β*, and *γ* are positively valued parameters used to adjust the importance of three corresponding factors. For simplification, *α*, *β*, and *γ* are kept as unity, and by default, *C*_3_ is kept as half of *C*_2_[53]. The simplified SSIM index is thus expressed as

(12) $$\begin{array}{c} {\text{SSIM}}_{\left(x,y\right)}=\frac{\left(2\mu_{x}\mu_{y}+C_{1}\right)\left(2\sigma_{x}\sigma_{y}+C_{2}\right)}{\left(\mu_{x}^{2}+\mu_{y}^{2}+C_{1}\right)\left(\sigma_{x}^{2}+\sigma_{y}^{2}+C_{2}\right)}. \end{array}$$

The edge preservation index (EPI) indicates the amount of edges that are preserved in the test image [55, 56]. Edges are of great importance in medical imaging as they contain significant information such as tumor or lesion contour identification [55, 56]. The EPI between reference image *A* and test image *B* is determined as

(13) $$\begin{array}{c} \text{EPI}\left(A,B\right)=\frac{\sum \left(∆A-\mu_{∆A}\right)\left(∆B-\mu_{∆B}\right)}{\sqrt{\sum {\left(∆A-\mu_{∆A}\right)}^{2}{\left(∆B-\mu_{∆B}\right)}^{2}}}, \end{array}$$

where *∆A* and *∆B* are obtained from filtering *A* and *B* using a high-pass filter [55, 56], such as a simple 3 × 3 Laplacian operator-based spatial domain filter *H* given as

(14) $$\begin{array}{c} H_{xy}=\left\{\begin{array}{c} 1,\text{for }x,y=1,2,3\text{ and }x,y\neq 2\\ -\overset{3}{\underset{i,j=1}{\sum }}H_{xy}\text{ for }x=y=2 \end{array}\right\}. \end{array}$$

*μ*_*∆A*_ and *μ*_*∆B*_ represent the mean of *∆A* and *∆*B, respectively. The EPI values range between 0 and 1; the higher the value the better is the image quality [55, 56].

Sharpness index (SI) is a no-reference image quality metric based on the image Fourier phase spectrum, which contains important information such as image geometry and contour details [55, 57]. The sharpness index (SI) of a test image *B* is defined as

(15) $$\begin{array}{c} \text{SI}=-\log_{10}\emptyset \left(\frac{\mu -\text{TV }\left(B\right)}{\sigma }\right), \end{array}$$

where TV(*B*) refers to the total variation of image *B*, *μ* = *E*(TV(*B*)), *σ*^2^ = Var(TV(*B*)), and ∅ is the tail of the Gauss distribution [55, 57]. A higher SI value corresponds to better image quality.

Structural content (SC) is a full-reference image quality metric based on image structural similarity and spatial arrangement of pixels in an image [58, 59]. The structural content metric equals 1 when two same images are compared. Structural content for reference image *A* and test image *B* is determined as

(16) $$\begin{array}{c} \text{SC}\left(A,B\right)=\frac{\sum_{x=1}^{X}\sum_{y=1}^{Y}{\left[A\left(x,y\right)\right]}^{2}\;}{\sum_{x=0}^{X}\sum_{y=0}^{Y}{\left[B\left(x,y\right)\right]}^{2}}. \end{array}$$

Normalized absolute error (NAE) is a full-reference image quality metric measuring the statistical difference between the reference and test images [58, 60]. A lower NAE value corresponds to better image quality. NAE for reference image *A* and test image *B* is given as

(17) $$\begin{array}{c} \text{NAE}=\frac{\sum_{x=1}^{X}\sum_{y=1}^{Y}\left(\left|A\left(x,y\right)-B\left(x,y\right)\right|\right)}{\sum_{x=1}^{X}\sum_{y=1}^{Y}\left(\left|A\left(x,y\right)\right|\right)}. \end{array}$$

## 3. Results and Discussion

### 3.1. Shepp-Logan Phantom-Based Sparse-View FBP Implementation

#### 3.1.1. Experiment 1: Comparison of Proposed and Classical Shaping Mechanisms

**Figure 5:**
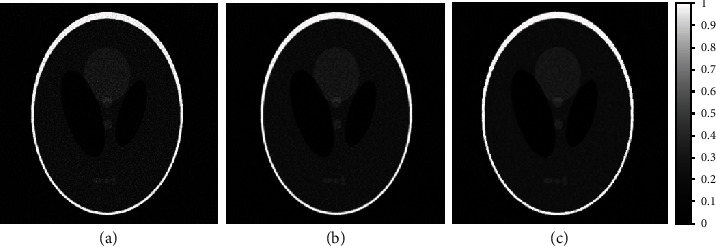
Comparison of sparse-view FBP reconstructed image with proposed RBS amoeba and classical amoeba schemes, using Gaussian filtering-based pilot image. (a) 2-degree angular sampling-based sparse-view FBP reconstruction. (b) Classical amoeba distance-based image denoising of sparse-view FBP. (c) Proposed RBS amoeba-based denoising using Gaussian-filtered pilot image.

To investigate the efficacy of the proposed RBS amoeba shaping mechanism, the proposed scheme is compared with the existing classical amoeba distance-based shaping. The two schemes are compared using Shepp-Logan-based sparse projections and a pilot image obtained from the Gaussian filtering. Mean filtering is then applied to the acquired amoeba shapes/kernels and evaluated based on the abovementioned image quality metrics, as shown in Figure 5.

The comparison of sparse-view FBP image with classical amoeba and proposed RBS amoeba schemes are shown in Figure 5. The sparse-view FBP reconstructed image is shown in Figure 5(a). The classical amoeba improves the image quality as shown in Figure 5(b). The RBS-based amoeba denoising with the Gaussian filter-based pilot is shown in Figure 5(c).

The proposed RBS amoeba shaping is visually better as compared to the classical amoeba scheme, with better SSIM, EPI, SI, SC, and NAE values, while presenting very similar quality in terms of PSNR and RMSE, as seen in Table 1.

#### 3.1.2. Experiment 2: Comparison of Proposed Wiener Filtering-Based RBS Amoeba Scheme with Classical Amoeba Scheme

**Figure 6:**
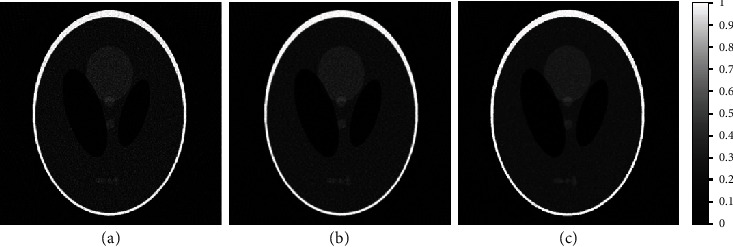
Comparison of sparse-view FBP reconstructed image with classical and proposed amoeba schemes. (a) 2-degree angular sampling-based sparse-view FBP reconstruction. (b) Classical amoeba filtering-based image denoising of (a). (c) Proposed scheme with RBS amoeba denoising of (a) using Wiener pilot image.

The proposed scheme, RBS amoeba with Wiener pilot, is compared with the classical amoeba scheme (Gaussian pilot), as shown in Figure 6. The 2-degree angular sampling-based FBP is shown in Figure 6(a). The classical amoeba-based enhanced image is shown in Figure 6(b), while the proposed amoeba scheme-based denoised image is shown in Figure 6(c).  Table 2 depicts that the proposed scheme presents superior results as compared to the classical amoeba scheme, using various image quality metrics.

**Figure 7:**
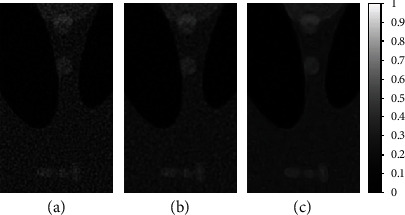
Comparison of enlarged ROI in sparse-view FBP reconstruction image with proposed amoeba and classical amoeba schemes. (a) 2-degree angular sampling-based sparse-view FBP reconstruction. (b) Classical amoeba distance-based image denoising of (a). (c) Proposed RBS amoeba denoising of (a) using Wiener pilot.

For better visualization and focused analysis of this comparison, Figure 7 presents an enlarged version of the results shown in Figure 6. The region of interest (ROI) is focused inside the phantom, such that the comparison does not include the streak artifacts outside the phantom (skull) boundary. The image quality is compared for the enlarged ROI only. The enlarged ROI of the FBP reconstructed image is shown in Figure 7(a), with the classical and proposed amoeba schemes shown in Figures 7(b) and 6(c), respectively. The proposed scheme is visually and statistically better than the classical amoeba scheme, as shown in Table 3. The focused ROI analysis demonstrates that the edges are more pronounced with enhanced contrast in the proposed scheme denoising, with better smoothening and noise suppression.

### 3.2. Real Clinical Head CT-Based Implementation

**Figure 8:**
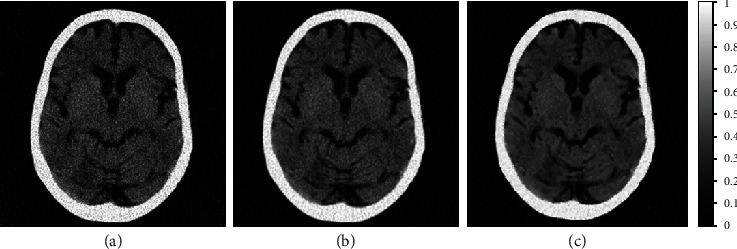
Comparison of sparse-view FBP reconstructed image with classical and proposed amoeba schemes, applied on clinically reconstructed CT image. (a) 2-degree angular sampling-based sparse-view FBP reconstruction. (b) Classical amoeba filtering-based image denoising of (a). (c) Proposed scheme with RBS amoeba denoising of (a) using Wiener pilot image.

The comparison of sparse-view FBP reconstruction of clinically reconstructed CT image, available at [52], with the classical and proposed amoeba denoising schemes, is shown in Figure 8. The sparse-view FBP reconstruction is shown in Figure 8(a). The classical amoeba (Gaussian pilot and amoeba distance) is shown in Figure 8(b), while the proposed scheme denoising is shown in Figure 8(c).

The proposed RBS amoeba shaping outperforms the classical amoeba scheme in terms of various image quality metrics, as shown in Table 4.

### 3.3. Robustness Comparison

**Figure 9:**
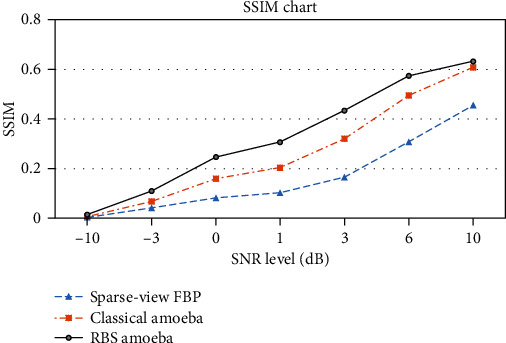
Robustness comparison of sparse-view FBP reconstruction with classical and proposed amoeba schemes, SSIM performance of the schemes at various noise levels in projection data.

**Figure 10:**
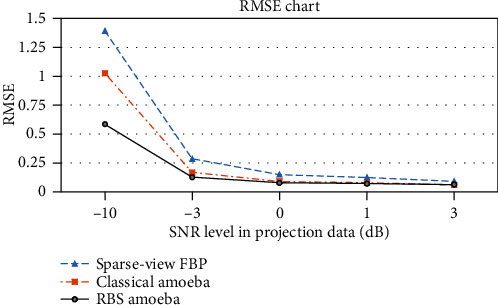
Robustness comparison of sparse-view FBP reconstruction with classical and proposed amoeba schemes, on the basis of RMSE performance of the schemes at various noise levels in projection data.

The robustness of the scheme was also tested by analyzing the image quality at various projection noise levels. Shepp-Logan phantom of size 512 × 512 was used for projection acquisition at 2-degree angular intervals. The FBP reconstruction quality was compared with classical amoeba and proposed Wiener pilot-based RBS amoeba denoising. The comparison demonstrates that the scheme is robust in the presence of noise, with both increased similarity index and lower error floors, as depicted in Figures 9 and 10, respectively.

### 3.4. Comparison of Schemes at Various Projection View Sampling

**Figure 11:**
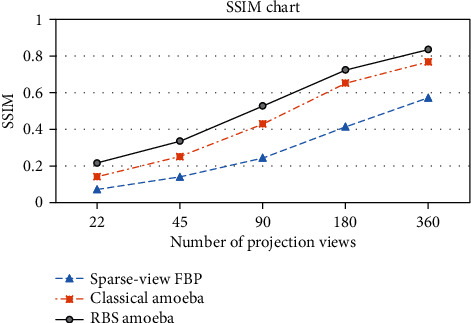
Comparison of sparse-view FBP reconstruction with classical and proposed amoeba schemes, SSIM performance of the schemes using FBP reconstructions at various projection view sampling.

The effectiveness of the scheme was also evaluated by analyzing the image quality at various projection view sampling. Shepp-Logan phantom of size 512 × 512 was used for FBP reconstruction, while the projection sampling ranges from 8-degree incremental angles to 0.5 degrees. The quality was compared with classical amoeba and proposed Wiener pilot-based RBS amoeba denoising. The comparison demonstrates that the scheme performs better even with a lesser number of projection views, as shown in Figure 11. Hence, the scheme presents an alternate radiation dose reduction method through the enhancement of sparse-view.

## 4. Discussion

The results demonstrate that the proposed RBS amoeba scheme has improved results as compared to the classical amoeba scheme. The sparseness in projections results in missing data; however, the human anatomy with symmetric and naturally contagious pixels serves as prior knowledge and enables the proposed scheme to perform better in this scenario. Therefore, the proposed scheme is a more suitable candidate for CT images. Moreover, the RBS scheme provides better contrast as compared to the classical amoeba distance-based denoising, as shown in enlarged ROI-based comparison.

The proposed scheme is more robust and performs significantly better than the classical amoeba filtering in the presence of projection noise. The scheme inherits significant image noise reduction, Gaussian, and otherwise, due to dependence on Wiener and Amoeba filtering [22, 26, 30]. The superiority of Wiener filter pilot over Gaussian filter pilot corresponds to lesser error floor in case of increased noise levels; while the similarity is increased mainly because of the region-based approach of the scheme matching the human anatomy. The proposed scheme provides better smoothening of the degraded image without intervening in the contour boundaries. The scheme provides an alternate method to reduce the radiation dose involved in CT, through enhancement of FBP reconstruction from a lesser number of projection views to a quality equivalent to more view data.

A limitation of the proposed scheme is its dependency on Otsu's multilevel thresholding method which makes it computational expensive and, therefore, is not appropriate for real-time applications. However, this can be mitigated by the use of advanced and high-speed processors, such as GP-GPU (general purpose graphical processor units), as the scheme has potential to run in parallel. Therefore, the future research directions may include scheme optimization for real-time applications and implementation on GP-GPU. Moreover, the adaptive nature of the proposed amoeba-based filtering indicates scheme implementation in many dynamic applications is worth investigating, signifying future application of the scheme on denoising of natural images, industrial-CT, and nondestructive testing (NDT) data.

## 5. Conclusion

This paper presents an efficient and novel postprocessing scheme for CT radiation dose reduction and enhancement of FBP reconstructed image from sparse-view noisy CT scans. In this work, a new type of amoeba filtering is presented, which is customized for CT images. Region-based segmentation (RBS) using multilevel thresholding was used in the amoeba kernel shaping, which is more effective in medical imaging applications as it is similar to the symmetric and region-based nature of the human body anatomy. The pilot image uses Wiener filter, which helps in noise suppression while keeping the edge and contour details required for amoeba shaping. The scheme is supported by computer simulations using fan-beam projections of clinically reconstructed and simulated head CT phantoms. The results demonstrate that the proposed Wiener filter-based RBS amoeba scheme is visually and statistically better than classical amoeba filtering for CT image, as evaluated using various image quality matrices. The presented scheme is more robust to noise in CT projections and effective for enhancing few-view reconstruction. In the future, the implementation of the scheme on more medical as well as industrial phantoms will be undertaken. The introduction of the Wiener filter-based RBS amoeba scheme makes way for a family of morphological, median, and other filters based on the presented framework. The algorithm has the potential to run in parallel; thus, implementation of the proposed scheme on GP-GPU will also be a possible future avenue.

## Figures and Tables

**Pseudocode 1:**
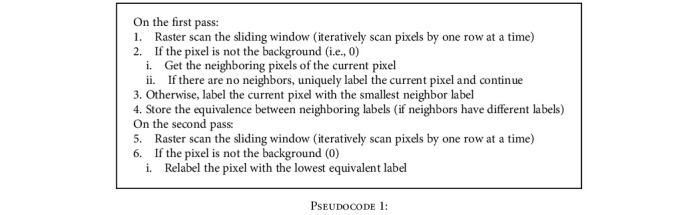
Pseudocode 1:

**Pseudocode 2:**
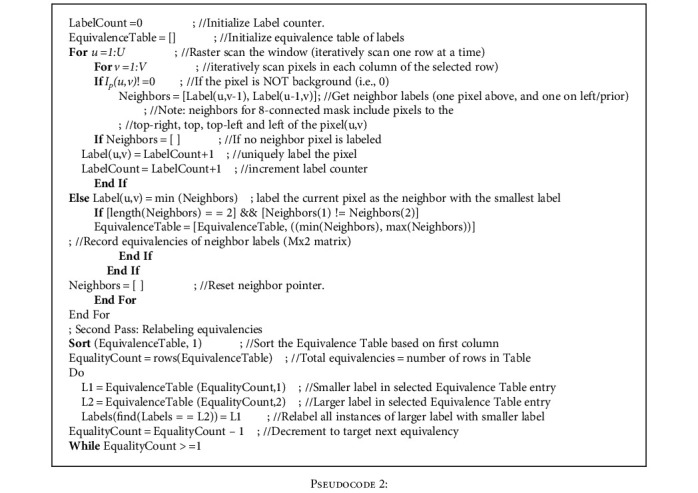
Pseudocode 2:

**Table 1:** Image quality comparison of proposed and classical shaping mechanisms.

Scheme name	RMSE	PSNR (dB)	SSIM	EPI	SI	SC	NAE
Sparse-view FBP	0.074	22.61	0.24	0.211	121	0.988	0.428
Amoeba distance (with Gaussian pilot)	0.059	24.56	0.42	0.253	332.8	0.990	0.286
RBS amoeba shaping (with Gaussian pilot)	0.059	24.51	0.52	0.261	1683.9	1.008	0.235

**Table 2:** Image quality comparison of the proposed Wiener filtering-based RBS amoeba scheme with the classical amoeba filtering scheme.

Scheme name	RMSE	PSNR	SSIM	EPI	SI	SC	NAE
Sparse-view FBP	0.074	22.61	0.24	0.211	121	0.988	0.428
Classical amoeba	0.059	24.56	0.42	0.253	332.8	0.990	0.286
Proposed RBS amoeba	0.052	25.56	0.58	0.376	1739.8	1.002	0.233

**Table 3:** Image quality comparison of schemes using enlarged ROI images.

Scheme name	RMSE	PSNR	SSIM	EPI	SI	SC	NAE
Sparse-view FBP	0.048	26.28	0.32	0.266	0.48	0.936	0.300
Classical amoeba	0.030	30.23	0.54	0.410	1.50	0.987	0.185
Proposed RBS amoeba	0.024	32.23	0.68	0.423	32.70	1.000	0.131

**Table 4:** Image quality comparison of the proposed and classical schemes in real clinical head CT-based implementation.

Scheme name	RMSE	PSNR	SSIM	EPI	SI	SC	NAE
Sparse-view FBP	0.085	21.32	0.22	0.211	5.75	0.959	0.290
Classical amoeba	0.053	25.46	0.37	0.210	40.17	0.993	0.179
Proposed RBS amoeba	0.047	26.52	0.44	0.289	350.3	1.002	0.142

## Data Availability

No data were used to support this study.

## Appendix

### A. Pseudoalgorithm for Connected Component Labeling

The pseudo algorithm to scan and segment connected regions is given below:

### B. Pseudocode for Connected Component Labeling

Pseudocode to scan and segment connected regions, using 4-connected component mask, is given below:
